# Supplementary figures and images for: Identification of modifier gene variants overrepresented in familial hypomagnesemia with hypercalciuria and nephrocalcinosis patients with a more aggressive renal phenotype
Source: PLoS Genet. 2025 Apr 2;21(4):e1011568. doi: 10.1371/journal.pgen.1011568 (PMC12005529; doi:10.1371/journal.pgen.1011568)

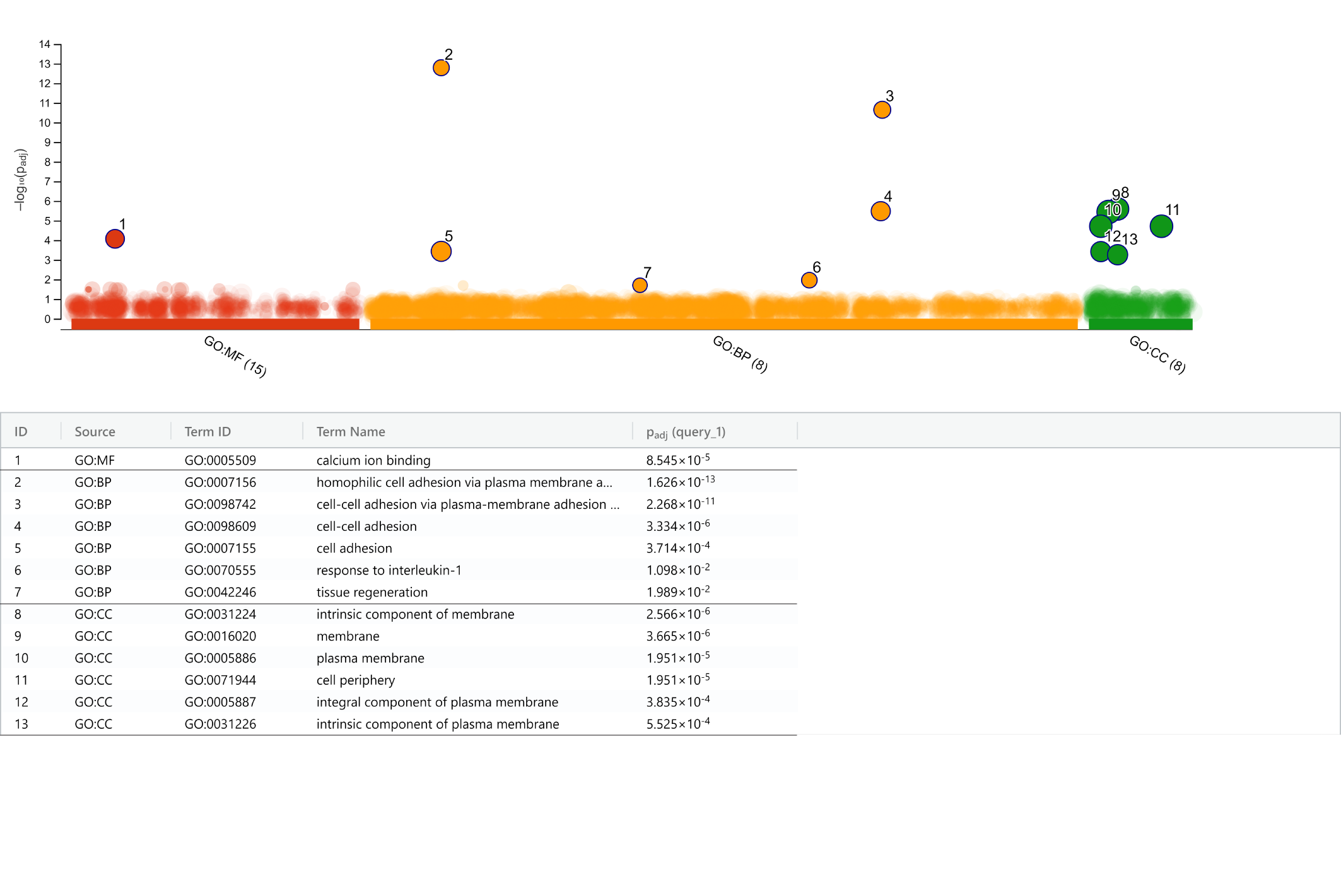

Supplement: S1 Fig — The most significant results for Gene Ontology (GO) are shown. GO molecular function (GO:MF); GO biological process (GO:BP). (TIF) [file pgen.1011568.s001.tif]
