# Supplementary material for: Identification of modifier gene variants overrepresented in familial hypomagnesemia with hypercalciuria and nephrocalcinosis patients with a more aggressive renal phenotype
Source: PLoS Genet. 2025 Apr 2;21(4):e1011568. doi: 10.1371/journal.pgen.1011568 (PMC12005529; doi:10.1371/journal.pgen.1011568)
Supplement: Attachment — Submitted filename: PGENETICS-D-24-00610_Point-to-point response_R2.pdf [file pgen.1011568.s011.pdf]

ADDITIONAL EDITOR COMMENTS:

The paper is much improved. One reviewer is still not satisfied, although states that the paper has improved. The main concern of this reviewer is the statistics which he / she states should be more stringent for this type of work which I do not agree, otherwise it would be impossible for ultra rare diseases to publish anything. The number of patients reported is the largest ever for this ultra rare disease (over 40). The authors nevertheless should indicate as a limitation that clinical decisions should not be made based on the data provided and that further cohorts should be studied. They also have to clarify when they use the value of p <0.05 and/or p <0.01.

Thanks very much for your response. We have indicated this specific limitation in the abstract and discussion and clarified the points regarding the p values in the new version of the manuscript.

RESPONSE TO REVIEWERS

Reviewer #2: The manuscript is improved, but the same methodological concerns remain, see below. I fully appreciate the difficulties of performing robust statistics in an ultrarare disorder, but does that really mean that we should burden these patients with potentially misleading and erroneous results? These results, once published, will almost certainly be picked up by patients, who may request testing for these results to inform important life decisions ("my unborn child has FHHNC and if it has risk variants for fast progression, I want to terminate that pregnancy"; or " I am thinking of going to university now, but if I am carrying variants for fast progression, I probably won't be able to conclude the studies because I have to start dialysis"). Their clinicians are then faced with trying to explain to these patients that these results are highly speculative, as the analysis bypassed robust statistical analysis. Or, worse, their clinicians may similarly take these results at face value as they are published in a respected peer-reviewed journal. Is the appropriate response to the problem of rare disorders really that we allow a 4.000-fold higher likelihood of false positive results?

The first thing we want to say is that we are not proposing any diagnostic kit or test with the results derived from this paper that could jeopardize anyone's life, but appreciate your comments and we have included a sentence in the abstract and discussion sections to prevent the patients from potential misleading erroneous results conducting to false expectations and frustration. Definitively, results presented in this manuscript are not intended to take medical or clinical decisions but rather to gain insights into the pathophysiology of this disease, thanks to our particular cohort of patients. As mentioned in the manuscript, we are studying a pediatric cohort that includes 45 affected Spanish patients from a population of 48.000.000 inhabitants. Given that the prevalence of the disease is 1 in 1 million, we have been able to recruit practically all the patients in Spain. This tremendous effort has been possible thanks to the contribution of pediatric nephrologist all over the country, the affected patients and their families and the patients' association HIPOFAM that has a long standing collaboration with us and are aware of the limitations of our research results.

The particularities of our cohort include:

i) 91% of the patients have mutations in the CLDN19 gene
ii) 70% of them are carrying the so-called Hispanic mutation in homozygosis (pG20D), and the rest of the patients in heterozygosis.
iii) All affected patients are pediatric with no co-morbidities and no renal injury or disease for other causes (diabetes, hypertension, drug toxicity, acute kidney injury for any cause, infection, etc). This point mutation is the only cause of their disease.
iv) The advantage of having severe vs moderate/slow affected siblings in the same family with the same pG20D CLDN19 gene mutation in homozygosis.

For all these reasons, we strongly believe that a cohort like this, and in general monogenic rare diseases cohorts, albeit their limitations, can shed light not only into the mechanisms affecting the disease under study but, likely, to other polygenic kidney diseases with associated co-morbidities, where it might be difficult even when higher number of patients and robust statistics would be possible.

Our research opens a window of opportunity and hope for patients that have an ultra-rare disease and for which no research could be ever possible, given their low number and lack of robust statistics.

We have initiated collaborations with French and Polish geneticists and pediatric nephrologists to obtain DNA samples and clinical information to further validate our results in other cohorts of patients. Moreover, we are going to study some of the most relevant variants in other renal diseases of unknown origin where patients underwent renal transplantation early in life.

1. Regarding comment 1: I did not want to imply that the authors added genes not identified in their initial association study. I simply wanted to summarise the candidate gene approach they took in that step, which, if I understand correctly, is still an appropriate summary. The key message here is that it is not an unbiased assessment of genetic variants, but a highly selected one, which obviously affects interpretation of the results. Did the authors decide on their selection strategy (which appears to be a] any gene expressed in the kidney and in selected categories of GWAS-catalogue, b] anything associated with eye and in selected categories of GWAS-catalogue, c] anything carrying the name claudin) a priori, or did they apply their "state-of-the art-knowledge" once they had the initial association results?

As stated in the manuscript and depicted in the workflow diagram, we initially identified associations through an unbiased assessment of genetic variants using SKAT-O. Subsequently, we applied a priori knowledge to prioritize genes and variants, aiming to maximize the biological relevance of our findings within the context of our study's scope and sample size. This integrated approach allowed us to address limitations related to sample size and highlight potential mechanisms warranting further investigation.

2. Line 139: Please detail, what percentile of predicted damaging variants the categories "moderate and high" would correspond to.

We have noted a mistake regarding the total number of variants and genes identified and have made the appropriate correction in the revised manuscript. In our analysis, we identified a total of 110,877 variants. Of these, 29,046 (26.2%) were classified as having

either high or moderate impact. Specifically, 1,019 variants (0.9%) were categorized as high impact, and 28,027 variants (25.3%) as moderate impact.

3. Lines 142 and 485: I am still confused by the statistics. In the rebuttal, the authors state that they now use FDR-corrected p-values, but in the results (line 142), they state that they still use $p<0.05$. And according to Methods (line 485), they now use a p-value of 0.01, instead of the previous 0.05: what is it now? And is a $p<0.01$ supposed to be the appropriate FDR correction when dealing with variants in ~20k genes? If so, based on which assumptions? Or is that an arbitrary level chosen by the authors? They state that it is "more stringent": More stringent than what? An FDR-corrected p-value? The accepted corrected p-value for exome-wide gene-based analysis is 2.5x10^-6: the "more stringent" value of 0.01 is 4000 times larger, meaning that the risk that their results are false-positive is 4000 times larger than what is generally accepted in the scientific community! Do the authors really want to take responsibility for such an increased risk that their results are misleading?

The low p-value the reviewer refers may make sense in large studies, but in smaller studies, the context and the biological mechanisms at play can provide valuable insights that may not be fully captured by traditional statistical measures. Therefore, while the p-value is a useful tool, it should not be the sole determinant of the validity of our results. We want to emphasize that, although we are including FDR adjusted p-values, we are merely indicating a direction rather than claiming to have identified a clear effect or suggesting that we will use this information for any clinical prediction.

4. Line 194: Again, a p-value of 0.05 is used, even though dealing with 175 variants: is that FDR-corrected?

Adjusted p-values for the variants discussed in the manuscript are provided in Supplementary Table 3. Given the challenges of achieving robust statistical power in ultra-rare diseases like FHHNC, we consider an FDR-adjusted p-value threshold of <0.15 acceptable for our exploratory analysis. We have highlighted the study's limitations and the need for further validation throughout the manuscript. In our revised version, we clarify that we prioritize variants with a nominal p-value <0.05 and FDR-adjusted $p <0.15$. Noteworthy two variants showed FDR-adjusted $p <0.05$, from those, the NFU1 variant, which shows the largest effect size and is our most promising candidate, has an FDR-adjusted p-value of 0.004.

Reviewer #3: I think this is an interesting and well-performed study. The authors correctly address what I find are the main limitations of the study: a limited number of patients and the lack of a sound validation cohort.
I also find the authors correctly address other reviewer's concerns and have adapted the text to them.

Thanks very much for your positive comments

Reviewer #4: The authors have adequately addressed the concerns that were raised by referees.

Thanks very much for your positive comments
