# Supplementary material for: Identification of modifier gene variants overrepresented in familial hypomagnesemia with hypercalciuria and nephrocalcinosis patients with a more aggressive renal phenotype
Source: PLoS Genet. 2025 Apr 2;21(4):e1011568. doi: 10.1371/journal.pgen.1011568 (PMC12005529; doi:10.1371/journal.pgen.1011568)
Supplement: Attachment — Submitted filename: PGENETICS-D-24-00610_Point-to-point response_R1.docx [file pgen.1011568.s010.docx]

**Editor’s comments:**

**The manuscript was fully evaluated at the editorial level and by independent peer reviewers. The reviewers appreciated the attention to an important problem, but raised some substantial concerns about the current manuscript. Based on the reviews, we will not be able to accept this version of the manuscript, but we would be willing to review a much-revised version. We cannot, of course, promise publication at that time. Specifically, reviewer 1 was concerned about the statistical analyses and the failure to correct for multiple testing. In addition, we were concerned about the lack of a validation cohort. Even though you did add new samples they could not actually validate as only one of the two phenotypes were added. We recognize that given the rarity of the disease this is difficult, but you will need to address both of these issues in any revised manuscript.**

First, we would like to note that the editorial office informed us of a confusion regarding the number of reviewers assigned to our manuscript. This was due to a duplicate account of one reviewer, resulting in one review being submitted twice. As advised in the email from the editorial office on October 8th, we will focus our revisions and responses on the comments provided by reviewers #2 through #4. We appreciate the editorial team's assistance in resolving this matter.

We sincerely appreciate the thorough evaluation of our manuscript and the valuable feedback provided by the reviewers. We understand the concerns raised and are committed to addressing them comprehensively in a revised version of our manuscript.

We acknowledge the importance of addressing multiple comparisons in our statistical analyses, as highlighted by Reviewer 2. In response, we have now included FDR-corrected p-values in the revised manuscript to ensure that readers are aware of the limitations in interpreting our WES analysis. This adjustment is crucial for maintaining transparency and rigor, particularly given the exploratory nature of our study. Additionally, we have revised the entire manuscript to temper our conclusions and explicitly state this limitation, emphasizing the need for caution in interpreting our results. We also fully understand the concerns about the limitations of our current validation approach. We recognize that the additional 15 patients we included do not constitute a complete validation cohort, particularly due to the lack of fast progressors. We acknowledge this as a significant limitation of our study. However, we must stress, as the editors also pointed out, the added challenge of working in the context of an ultra-rare disease like FHHNC, where patient cohorts are inherently small and replication studies are exceptionally difficult to conduct. The unique genetic landscape of our cohort, which exhibits a remarkable genetic homogeneity, combined with the comprehensive inclusion of all diagnosed FHHNC patients across Spain, provides a valuable platform for identifying potential genetic modifiers that may influence disease progression and phenotypic variability. We understand that scientific discovery is an iterative process, where hypotheses are tested and refined over time. Our goal is to provide a foundation for future research while acknowledging the limitations inherent in studying rare diseases. Indeed, we are currently validating promising candidates using iPSC-derived 3D renal organoid models from sibling patients with divergent phenotypes and harboring distinct candidate risk variants identified in this study, and we have initiated international collaborations (in France and Poland) to replicate our findings in two separate cohorts. However, this process is challenging and time-consuming due to the ultra-rare nature of FHHNC, with an estimated worldwide prevalence of fewer than 1 per million. The geographical dispersion of patients, limited awareness of the disease, and the small patient pool pose significant hurdles in assembling a sufficiently large cohort for robust replication. Despite the limitations, these efforts underscore our commitment to rigorous scientific investigation in a field with significant challenges, including ethical constraints on obtaining renal biopsies in pediatric patients and a lack of appropriate in vitro models for the thick ascending loop of Henle.

In line with current discussions in the scientific community about the interpretation of genetic data, we are mindful of the potential for overstatement of findings. Our approach carefully considers the balance between exploratory research and rigorous validation, similar to debates surrounding genotype-phenotype relationships in other complex diseases. We aim to contribute to this ongoing dialogue by ensuring that our terminology and claims are precise and supported by robust evidence. We are committed to transparency and rigor in all aspects of our work, aligning with best practices in human genetic studies and appreciate the opportunity to revise our manuscript to address these important issues.

**Reviewer #2:**

**The authors present a study on potential modifier variants for FHHNC. They perform WES in a cohort of 30 patients with FHHNC which they stratify by extremes with regards to kidney disease progression and identify 29870 variants in 11240 genes. Of these, 1068 genes were significantly associated with progression. They select from these 165 genes based on expression in the kidney and select further from these based on their representation in the GWAS catalogue as associated with phenotypic traits they consider relevant. They then add in genes from the GWAS catalogue associated with ocular impairment resulting in 44 candidate genes. They then add back in a few genes even though they are not expressed in the kidney (but with relevant GWAS catalogue association) and one because they like the name (it is a Claudin), ending up with 50 candidate gens containing 175 variants. These 175 variants were then individually tested for association with renal progression, of which 19 made the cut.**

We appreciate the reviewer’s feedback and welcome the opportunity to clarify our methodology. Our variant selection process was entirely based on our WES analysis, without introducing genes from external sources such as the GWAS catalogue or any database purely based on their association or relevance to our study. All identified genes showed statistically significant association with renal progression using the SKAT-O method. While we acknowledge the importance of correcting for multiple comparisons, due to power limitations inherent in studying an ultra-rare disease like FHHNC, we applied a more stringent threshold (p<0.01) instead of FDR correction, as explained in the manuscript. It's important to note that while our statistical power is limited due to the ultra-rare nature of FHHNC, our study holds significant value as it encompasses the entire FHHNC patient population in Spain. This unique cohort provides unprecedented insights into the genetic landscape of FHHNC, despite the challenges posed by small sample sizes which are well recognized (Nat Rev Methods Primers. 2022 Mar 10;2(1):s43586-022-00100-2.)

Contrary to the reviewer's interpretation, we did not add genes from the GWAS catalogue associated with ocular impairment. Instead, we combined the statistical criterion (p<0.01) with a systematic prioritization strategy, considering the relevance of the variants obtained to FHHNC-related phenotypic traits including ocular impairment. All genes in our analysis, including those associated with ocular manifestations, were identified through our WES analysis of the FHHNC cohort. The GWAS catalogue was used as a reference to assess the potential relevance of these genes to FHHNC-related traits, but it was NOT a source for adding new genes to our analysis. Genes not expressed in the kidney were included to explore potential influences from other tissues on FHHNC progression. This comprehensive approach allows us to investigate a broader range of potential genetic modifiers that may impact disease outcomes.

Regarding the claudin family, their inclusion was based on their well-established role in tight junction biology and potential involvement in kidney pathophysiology. Mutations in CLDN16 and CLDN19 are the primary cause of FHHNC, but other claudins like CLDN10 and CLDN14 are known to interact with these proteins and influence renal ion transport. CLDN10 forms paracellular cation channels in the thick ascending limb of Henle's loop, where it functions together with CLDN16 and CLDN19 to maintain renal ionic homeostasis (Genes (Basel). 2020 Mar 10;11(3):290). CLDN14, on the other hand, can bind to CLDN16 and inhibit the cation-selective paracellular permeability of CLDN16-CLDN19 heteromeric channels (PNAS. 2016 Dec 27;114(2):E219–E227; Genes (Basel). 2020 Mar 10;11(3):290). Our study identified variants in CLDN8 and CLDN17 which we prioritized as, being members of the claudin family, they could potentially contribute to FHHNC pathophysiology through related mechanisms, albeit with more limited available information. While the specific roles of CLDN8 and CLDN17 in renal physiology are less well-characterized than those of CLDN16, CLDN19, CLDN14 and CLDN10, they may be involved in potentially modifying FHHNC progression by altering the function of CLDN16-CLDN19 complexes or by compensatory paracellular transport mechanisms. CLDN8, for instance, has been implicated in the regulation of paracellular permeability to sodium and water in the distal nephron (Int J Mol Sci. 2019 Dec 28;21(1):221). CLDN17, though less studied in the context of renal function, is expressed in the kidney and could potentially modulate paracellular ion transport. Indeed, CLDN17 plays an important role in kidney function, particularly in regulating electrolyte balance and reactive oxygen species (ROS) levels (Cells. 2022 May 29;11(11):1782). By prioritizing the variants identified in these claudins in our analysis, we aim to explore broader mechanisms of tight junction dysfunction that may influence FHHNC outcomes in a multi-gene context. This approach allows us to investigate potential modifier effects on disease progression, even in the absence of direct interactions with CLDN16 and CLDN19.

We have revised the whole manuscript to incorporate these clarifications, aiming to provide a more comprehensive and transparent explanation of our methodology. We hope this clarification together with our response to other major points raised below addresses the reviewer's concerns and demonstrates the scientific rationale behind our methodological choices. We are happy to provide further details if needed.

**Major**

**1. Overall, this is a somewhat confusing paper. The selection of candidate genes and variants appears quite haphazard. A flow diagram would help to clarify the individual steps. Were the added in genes associated with ocular phenotype used for renal progression analysis? Analysed separately?**

Following the reviewer’s suggestion, we have included a flow diagram in the manuscript to illustrate each step of our workflow. This visual aid should help readers better understand our systematic approach to gene and variant selection. We thank the reviewer for this suggestion.

As stated above, we would like to emphasize that we did not add genes from external sources such as the GWAS catalogue. All genes in our analysis, including those associated with ocular phenotypes, were identified through our WES analysis of the FHHNC cohort. Our approach combined a statistical criterion (p<0.01) with a systematic prioritization strategy that considered the relevance of variants to FHHNC-related phenotypic traits, including both renal and ocular manifestations. To address the reviewer's specific question about the analysis of genes associated with ocular phenotypes, these genes were indeed included in our renal progression analysis as we aimed to explore potential pleiotropic effects or shared pathways between renal and ocular manifestations in FHHNC. Additionally, we are currently conducting a separate analysis of our WES results by stratifying our patient cohort into two groups based on ocular phenotype. This ongoing work aims to identify candidate phenotype-modifying variants specifically associated with the prototypical ocular manifestations observed in FHHNC patients. We anticipate that this analysis will provide further insights into the genetic factors influencing the diverse clinical presentations of FHHNC.

We hope this clarification, along with the newly added flow diagram, addresses the reviewer's concerns about the perceived haphazardness of our gene selection process.

**2. The statistics are quite confusing. P-values are considered significant sometimes at <0.05, sometimes <0.01, sometimes <0.25 and it is not clear how these levels were derived**.

We appreciate the reviewer's attention to our statistical approach and apologize for any confusion. To clarify, our primary significance threshold was consistently p<0.05, which is a widely accepted cutoff to differentiate between meaningful findings and random chance. We applied a more stringent cutoff of p<0.01 for the WES and SKAT-O analyses to reduce the likelihood of false positives, as these results did not pass multiple comparison correction due to the limited sample size inherent in studying an ultra-rare disease. The value of <0.25 that the reviewer noted is not a p-value, but an adjusted p-value (after correcting for multiple comparisons) used exclusively in the enrichment analysis. The enrichment analysis used a less stringent threshold to capture a broader range of potentially relevant biological processes, as is common practice in exploratory functional analyses (Nat Commun 2019,10:4274; Nat Commun 2024,15:3764; Nature Biomedical Engineering 2024,8:890-908). This analysis was performed only to functionally annotate the candidate genes and did not influence our prioritization pipeline.

We have revised the text in the Methods section to explicitly state these thresholds and their rationale, ensuring clarity for readers. We thank the reviewer for highlighting this need for clarification.

**3. Most importantly: the fact that p-values were not corrected for multiple comparisons makes the whole statistics essentially useless. They state that they mitigate the risk of false-positives by using “state-of-the-art knowledge of the disease”, but why do statistics then in the first place? Who needs statistics if you have “state-of-the-art knowledge of the disease”?**

We acknowledge the importance of correcting for multiple comparisons and agree that failing to do so increases the risk of false positives. In response to the reviewer’s concerns, we have now included FDR-corrected p-values for our WES analysis in the revised manuscript. This addition should provide a more comprehensive view of our results and allow readers to interpret them with appropriate caution. However, we respectfully disagree that this renders our statistics "essentially useless.", and we would like to clarify our approach and rationale.

FHHNC's ultra-rare nature presents unique challenges in achieving robust statistical power. However, our cohort, though small, offers an unprecedented opportunity for a comprehensive study of this disease. It encompasses, to our knowledge, all diagnosed FHHNC patients in Spain that we have clinically phenotyped and followed up since they were initially diagnosed until present. This cohort exhibits remarkable genetic homogeneity, with >90% of patients harboring mutations in *CLDN19*, and among these, over 70% carrying the same homozygous mutation (p.G20D). Importantly, our previous work has demonstrated significant phenotypic variability even among patients with identical genotypes, including siblings. This variability is particularly evident in the rate of progression to end-stage renal disease and the severity of ocular involvement, suggesting the influence of additional genetic or environmental factors beyond the primary *CLDN19* mutation. This genetic uniformity, combined with the comprehensive inclusion of all diagnosed FHHNC patients across Spain, provides a unique and highly valuable platform for investigating FHHNC. This cohort is particularly well-suited for identifying potential genetic modifiers that influence disease progression and phenotypic variability, offering insights that may be applicable beyond our specific patient group. We initially employed a more stringent p-value threshold (p<0.01) as an attempt to mitigate false positives given our limited sample size. However, we recognize that this approach alone is not sufficient to fully address the multiple comparisons problem. Our methodology integrates statistical analysis with current biological knowledge, which is a common approach in high-throughput -omics research in order to prioritize candidates, and is especially valuable for rare diseases where statistical power may be limited due to small sample sizes. The prioritization pipeline we applied combines statistical significance with biological plausibility, using public databases like GWAS and GTEX to contextualize and prioritize the variants we identified through WES. As clarified above, we did not introduce new variants from these databases but used them to assess the relevance of our findings. We acknowledge the limitations, but our study still provides valuable foundational work for understanding genetic modifiers in FHHNC.

We have aimed to be transparent about our methods and their rationale throughout the manuscript. While we acknowledge that our approach is not without limitations, we believe it offers meaningful insights into FHHNC and opens new avenues for research in this challenging field. We remain open to further discussion and welcome any additional suggestions for improvement.

**4. Presumably, the p-value in the Fisher’s exact test for the individual variant association was also not corrected for multiple comparisons?**

To address the reviewer’s concern, we have included FDR-corrected p-values corresponding to the Fisher’s exact test in the revised manuscript.

**5. Line 203: I am very confused by this statement. These variants were selected because of their association with renal progression and then it is presented as a result that these variants are overrepresented in “fast progressers”? Isn’t that circular logic? Same for line 228.**

Our approach may, indeed, initially appear circular. We will clarify the strategy used to address this concern.

Our process begins with an initial identification of variants associated with renal progression based on our WES analysis and statistical criteria. We then prioritize these variants considering their biological plausibility and relevance to FHHNC-related traits. The overrepresentation analysis in 'fast progressors' serves as an independent validation step for our prioritized variants, rather than a circular confirmation of our initial selection. Interestingly, not all initially prioritized variants were found to be overrepresented in the 'fast progressor' cohort. This discrepancy, far from being a weakness, actually demonstrates the value of our multi-step approach. It helps us to identify potential false positives from our initial selection and allows us to refine our focus on variants that consistently show association with faster renal failure progression across different analytical approaches. This process of initial identification, prioritization, and subsequent validation helped us navigate the challenges of limited sample sizes and the complexity of genetic influences on disease progression.

We thank the reviewer for highlighting this need for clarification. In response, we have revised the text in the manuscript to more clearly explain this step-wise process and its rationale, ensuring that readers can fully understand and appreciate the robustness of our methodology.

**6. Given the potential of the “winner’s curse”, confirmation of the results from the discovery cohort in a validation cohort is essential. The fact that the validation cohort contains no “fast progressers” makes it unsuitable: How can you validate risk factors for fast progression in a cohort without fast progression? The fact that the risk factors are absent does not exclude the winner’s curse.**

We appreciate the reviewer's insightful comment regarding the potential 'winner's curse' and the importance of validation. We acknowledge that our validation cohort's composition is not ideal for confirming risk factors associated with fast progression. However, we believe our approach still provides valuable insights and mitigates some concerns about false positives.

We performed a technical validation of the identified variants using direct SNP genotyping by qPCR. This step confirms that our findings are not sequencing artifacts, adding confidence to the initial results. While our second cohort unfortunately lacks fast progressors, it still adds value to our results. The underrepresentation of risk variants associated with the more aggressive phenotype in this cohort of less aggressive cases supports our initial findings. This negative correlation, although not as strong as a positive replication, provides additional evidence for the specificity of these variants to the fast phenotype. Given the ultra-rare nature of FHHNC, assembling large cohorts with balanced phenotypes is extremely challenging. Our approach represents a pragmatic effort to validate our findings within these constraints. We have revised the discussion section in the manuscript to further acknowledge the limitations of our current validation approach and are actively working on expanding our collaborations to include more diverse FHHNC cohorts for future studies.

**minor**

**1. Abstract, line 38: not clear which gene the common variant is located in: CLDN16? Or 19?**

This has been corrected in the revised version.

**2. Line 125: explain GATK**

This has been corrected in the revised version.

**3. Line 127: explain SKAT-O**

This has been corrected in the revised version.

**4. Line 131: explain GO-BP and GO-CC**

This has been corrected in the revised version.

**Reviewer #3:**

**This is a very interesting and novel approach to the genetic risk factors of having a severe phenotype in patients with FHHNC. It is especially interesting taking into account that a high number of the patients of the cohort exhibit the same p.G20D variant, rendering a high genetic similarity that allows comparison between patients.**

**The main limitations of the study are, in my opinion, the low number of patients (specifically the low number of FAST patients), but this is understandable taking into account it is an ultra-rare disease. I find the authors discuss this point appropriately in their manuscript.**

**In addition, the genetic risk factors found in the FAST patients would need to be validated in other larger cohorts, as the validation cohort of 15 patients is probably not enough (again, especially the FAST patients). I find the authors also manifest in their manuscript this limitation point.**

**I have no other comments to the manuscript.**

We sincerely appreciate the time and effort Reviewer #3 has dedicated to evaluating our manuscript. We are grateful for their positive assessment of our novel approach to identifying genetic risk factors for severe phenotypes in FHHNC patients.

We thank the reviewer for recognizing the unique value of our cohort, particularly the high genetic similarity due to the prevalence of the p.G20D variant, which indeed provides a robust foundation for comparing phenotypic differences among patients. The reviewer's insightful comments and overall positive evaluation are really encouraging. Their feedback reinforces the potential impact of our work while appropriately contextualizing its limitations. We are committed to addressing these limitations in future research and appreciate the reviewer's support for our ongoing efforts in this challenging field.

Indeed, we are actively working to expand our study by recruiting additional FHHNC patients from Poland and France for replication. However, this process is challenging and time-consuming due to the ultra-rare nature of FHHNC. Despite these obstacles, we remain committed to validating our findings in a wider, international cohort, recognizing that such efforts are crucial for advancing our understanding of this rare disease.

Thank you again for the constructive and supportive review.

**Reviewer #4:**

**The manuscript by Vall-Palomar et al is titled “Identification of modifier gene variants overrepresented in familial hypomagnesemia with hypercalciuria and nephrocalcinosis patients with a more aggressive renal phenotype” and studies individuals with familial hypomagnesemia with hypercalciuria and nephrocalcinosis (FHHNC) which is caused by loss-of-function mutations in CLDN16 and CLDN19. The study examines the phenotypic variability in a mutation which is the most prevalent mutation in Spain. Patients were stratified according to their estimated glomerular filtration rate annual decline. Whole exome sequencing was obtained to find candidate phenotype-modifier genes.**

**The authors report the identification of 19 putative modifier gene variants associated with a higher risk of developing a more aggressive renal phenotype.**

**The authors specifically report the identification of a panel of genetic variants within novel candidate modifier genes which could be utilized for estimating the risk of developing early kidney failure in this disease.**

**The studies are well designed and well executed. I have few minor concerns.**

**1. There are several major limitations with these studies, but the authors are aware of them and have discussed these limitations as they pertain to the impact of specific variants in NFU1, DMD, HPS5, CLDN8 and CLDN17 on faster decline in kidney function. They need to expand on these limitations.**

We sincerely thank Reviewer #4 for their thorough evaluation of our manuscript and their positive assessment of our study design and execution. We appreciate their recognition of the potential impact of our work in identifying genetic variants that could help estimate the risk of early kidney failure in FHHNC patients.

We acknowledge the reviewer's comments regarding the limitations of our study and agree that these warrant further discussion. In response, we have added a more comprehensive discussion of these limitations in the revised manuscript. This includes addressing the small sample size inherent to studying an ultra-rare disease, the challenges of establishing causality for modifier genes, and the need for functional studies to confirm the biological impact of these variants.

**2. The authors would like to discuss the possible role of variants in SLC17A1 and SLC9A3 and their association with enhanced progression to renal failure in FHHNC. This referee was not aware of any studies pointing to the localization of the urate transporter SLC17A1 in the thick ascending limb.**

We thank the reviewer for bringing to our attention the lack of evidence for SLC17A1 localization in the thick ascending limb. We have revised our discussion to include current knowledge of SLC17A1 and SLC9A3 localization and potential roles in kidney function. We now focus on their known functions in other nephron segments and discuss how alterations in these transporters might indirectly affect the progression of renal failure in FHHNC.

We have made these revisions to provide a more balanced and accurate interpretation of our findings. We appreciate the reviewer's insightful comments, which have helped improve the clarity and scientific rigor of our work. We are grateful for their positive evaluation and their constructive feedback.
